# Moving towards on-site detection of Shiga toxin-producing *Escherichia coli* in ready-to-eat leafy greens

**DOI:** 10.1016/j.crfs.2024.100716

**Published:** 2024-03-07

**Authors:** Ana Costa-Ribeiro, Alexandre Lamas, Azucena Mora, Marta Prado, Alejandro Garrido-Maestu

**Affiliations:** a International Iberian Nanotechnology Laboratory, Av. Mestre José Veiga s/n, 4715-330, Braga, Portugal; b Department of Biochemistry, Genetics and Immunology, University of Vigo, 36310, Vigo, Spain; c Food Hygiene, Inspection and Control Laboratory (Lhica), Department of Analytical Chemistry, Nutrition, and Bromatology, Veterinary School, Campus Terra, Universidade de Santiago de Compostela (USC), 27002, Lugo, Spain; d Laboratorio de Referencia de *E. coli* (LREC), Dpto. de Microbioloxía e Parasitoloxía, Facultade de Veterinaria, Universidade de Santiago de Compostela (USC), Lugo, Spain; e Instituto de Investigación Sanitaria de Santiago de Compostela (IDIS), Santiago, Spain; f Laboratory of Microbiology and Technology of Marine Products (MicroTEC), Instituto de Investigaciones Marinas (IIM), CSIC, Eduardo Cabello, 6, 36208, Vigo, Spain

**Keywords:** STEC, Shiga toxin-producing *E. coli*, *stx*1, *stx*2, Point-of-care, Loop-mediated isothermal amplification, Colorimetric detection, Naked-eye, Glass milk

## Abstract

Rapid identification of Shiga toxin-producing *Escherichia coli*, or STEC, is of utmost importance to assure the innocuousness of the foodstuffs. STEC have been implicated in outbreaks associated with different types of foods however, among them, ready-to-eat (RTE) vegetables are particularly problematic as they are consumed raw, and are rich in compounds that inhibit DNA-based detection methods such as qPCR. In the present study a novel method based on Loop-mediated isothermal amplification (LAMP) to overcome the limitations associated with current molecular methods for the detection of STEC in RTE vegetables targeting *stx*1 and *stx*2 genes. In this sense, LAMP demonstrated to be more robust against inhibitory substances in food. In this study, a comprehensive enrichment protocol was combined with four inexpensive DNA extraction protocols. The one based on silica purification enhanced the performance of the method, therefore it was selected for its implementation in the final method. Additionally, three different detection chemistries were compared, namely real-time fluorescence detection, and two end-point colorimetric strategies, one based on the addition of SYBR Green, and the other based on a commercial colorimetric master mix. After optimization, all three chemistries demonstrated suitable for the detection of STEC in spiked RTE salad samples, as it was possible to reach a LOD50 of 0.9, 1.4, and 7.0 CFU/25 g for the real-time, SYBR and CC LAMP assays respectively. All the performance parameters reached values higher than 90 %, when compared to a reference method based on multiplex qPCR. More specifically, the analytical sensitivity was 100, 90.0 and 100 % for real-time, SYBR and CC LAMP respectively, the specificity 100 % for all three assays, and accuracy 100, 96 and 100 %. Finally, a high degree of concordance was also obtained (1, 0.92 and 1 respectively). Considering the current technological advances, the method reported, using any of the three detection strategies, demonstrated suitable for their implementation in decentralized settings, with low equipment resources.

## Highlights

• A novel STEC LAMP assay was developed for the detection of *stx*1 and *stx*2.
• Three detection strategies were evaluated, real-time fluorescence acquisition and two naked-eye colorimetric ones.
• Four simple DNA extraction and purification protocols suitable for point-of-care testing were compared.
• The “glass milk” protocol demonstrated to be the best option considering complexity, cost, turnaround time and performance.
• An LOD50 <10 CFU/ 25 g, with high diagnostic sensitivity, specificity and accuracy in spiked RTE salad samples was reached.

## Graphical abstract

**Figure:**
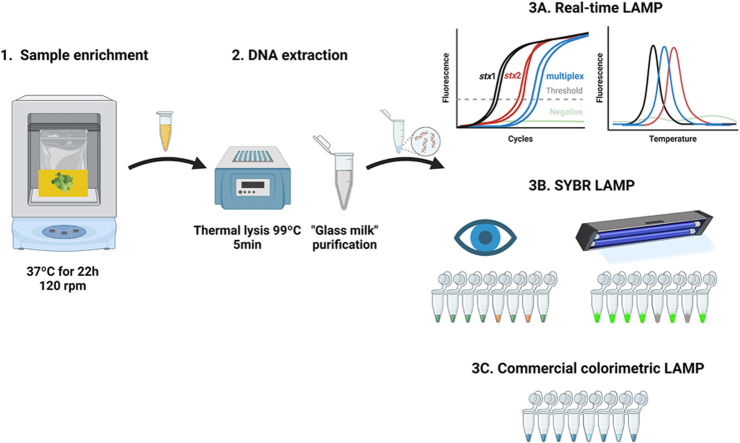

## 1 Introduction

Shiga toxin-producing *E. coli* (STEC) are among the most important foodborne pathogens worldwide, and the fourth most reported zoonosis in humans according to the latest European Union surveillance report (EFSA and ECDC, 2022). STEC infections have been linked to the consumption of different types of foods such as milk (Martin and Beutin, 2011), meat (Ethelberg et al., 2009), vegetables (Bottichio et al., 2020; Soon et al., 2013) among others (Erickson and Doyle, 2007; Mora et al., 2012). Out of these, ready-to-eat vegetables (RTE) are of particular concern as they are mainly consumed raw, thus rapid methods to better assure the innocuousness of these products are needed. DNA amplification-based methods have already demonstrated a suitable alternative to classical, culture-based methods furthermore, some new ISO standards already implement this type of techniques. For instance, ISO 13136 is based on the screening for *stx*1, *stx*2, and *eae* as well as serogroup-specific genes to determine the presence of STEC in foods by qPCR (ISO/TS, 2012). Unfortunately, these types of methods rely on expensive equipment and highly skilled personnel. Contrary to these, isothermal DNA amplification techniques, such as Loop-mediated isothermal amplification (LAMP), described by Notomi et al. (2000) can perform this task at one single temperature, typically 60–65 °C. In addition to this, LAMP is compatible with many different chemicals which allow for real-time amplification monitoring, end-point naked eye detection, or even both (Alejandro Garrido-Maestu et al., 2022; Mori et al., 2004). These features make LAMP an attractive technique for the development of Point-Of-Care (POC) assays, easily implementable in miniaturized devices (Kaygusuz et al., 2019; Moore et al., 2021; Safavieh et al., 2016). When focusing on the specific situation of vegetable samples, LAMP presents an added value over PCR/qPCR-based assays, and this is the fact that it is generally more resistant to the presence of typical inhibitors (Yang et al., 2014) such as chlorophylls and polysaccharides (Rossen et al., 1992; Schrader et al., 2012).

As it may be observed from what has been stated, LAMP seems like an ideal way to go for rapidly and accurately controlling STEC on-site, potentially even in a farm, or low-income areas, where limited laboratory resources and technical equipment are available. However, one limitation remains where not much effort has been put on, this is the sample preparation step, namely the potential need for an enrichment step and a suitable DNA extraction protocol to assure the absence of inhibitors and the recovery of enough amplifiable DNA. This is a common trend as already highlighted by Brehm-Stecher et al. (2009). Considering the existing limitations, the focus, and novelty, of the current study was to develop a novel STEC LAMP assay based on different detection chemistries to determine their suitability for decentralized implementation and to provide a comprehensive sample treatment protocol, including the enrichment and DNA extraction protocols also suitable for decentralized analyses. Due to the inherent problematic of STEC in RTE vegetables, the new method was tested in this food commodity. The methods demonstrated capable of overcoming the existing limitations of the studies previously reported for point-of-need (PON) application.

## 2 Materials and methods

### 2.1 Bacterial strains

STEC strain AMC 76, supplied by the Institute of Applied Microbiology – ASMECRUZ was selected as the reference microorganism for the optimization and evaluation of the newly designed LAMP assays. This strain was previously characterized, and it is known to bear *stx*1 and *stx*2 genes (Costa-Ribeiro et al., 2023a, 2023b). Additionally, this same bacterium was used for the inoculation experiments. Fresh cultures were prepared by resuspending one single colony in 4 mL of Nutrient Broth (NB, Biokar Diagnostics S.A., Allonne, France) and the suspension was incubated at 37 °C overnight. The following day, one hundred-fold serial dilutions were performed using fresh NB as the diluent, and these were plated on Tryptic Soy Agar (TSA, Biokar Diagnostics S.A., Allonne, France). The plates were incubated at 37 °C overnight to determine the concentration of viable AMC 76 spiked in the inoculation experiments.

ChromAgar™ STEC (CHROMagar Microbiology, Paris, France), from now on “Chrom”, was used for the confirmation of the results obtained by LAMP and qPCR. To this end, after the enrichment step detailed below, a loopful was streaked on the surface of the agar, the plates were incubated at 37 °C overnight and screened for typical mauve colonies on the following day.

The broth selected for the enrichment of STEC in RTE salad samples was modified Tryptone Soy Broth (mTSB, Biokar Diagnostics S.A., Allonne, France) supplemented with 16 mg/L of novobiocin as recommended by the ISO standard (ISO/TS, 2012), from now on “mTSBn”.

### 2.2 Sample processing

To 25 g of RTE salad, 50 mL of mTSBn were added. The corresponding concentration of freshly prepared AMC 76 bacterial culture as previously detailed in M&M 2.1, was added and homogenized for 30 s in a Stomacher 400 Circulator (Seward Limited, West Sussex, UK). The samples were incubated at 37 °C for 22 h at 120 rpm. After enrichment, 1 mL was taken for DNA extraction, and a loopful was streaked on Chrom.

### 2.3 DNA extraction

Four different DNA extraction protocols were compared for which three aliquots of the same sample were taken. The comparison was based on total DNA concentration extracted measured in a Qubit™4 Fluorometer (Invitrogen™, Carlsbad, CA, USA), its quality based on 260/280 and 260/230 absorbance ratios, measured in a NanoVue™ Plus Spectrophotometer (GE Healthcare Europe GmbH), and the overall performance in real-time LAMP considering the Cq obtained as detailed below. The four protocols were 1) direct thermal lysis, 2) chelex, 3) thermal lysis with magnetic beads purification, and 4) thermal lysis with “glass milk” purification. All four protocols started by centrifuging 1 mL of the enriched sample at 16000×*g* for 2 min. The supernatant was discarded, and the bacterial pellet was used for DNA extraction. At the end of each protocol, the supernatants containing the DNA were transferred to clean tubes, and stored at 4 °C until needed.

#### 2.3.1 Direct thermal lysis

The pellet was resuspended in 100 μL of nuclease-free water (Thermo Fisher Scientific, Inc., Waltham, MA, US) and heated at 99 °C for 5 min at 1400 rpm in a Thermomixer comfort, from now on thermomixer (Eppendorf AG, Germany). Bacterial debris, as well as food leftovers, were precipitated by centrifuging again at 16000×*g* for 2 min.

#### 2.3.2 Chelex

The pellet was resuspended in 100 μL of 6 % Chelex®100 (Bio-Rad Laboratories, Inc., USA). The suspension was incubated for 15 min at 56 °C and 1400 rpm, and subsequently heated at 99 °C for 8 min again at 1400 rpm. Both incubation steps were performed in a thermomixer. Finally, the samples were centrifuged at 16000×*g* for 2 min to precipitate food leftovers, cellular debris, and the resin.

#### 2.3.3 Thermal lysis with magnetic beads purification

The pellet was treated as in M&M 2.3.1 but, after the lysis, instead of centrifuging, 100 μL of magnetic beads Sera-Mag Select (Cytiva Europe GmbH, Cornellá de Llobregat, Spain) were added. The mixture was incubated for 5 min at room temperature in a Mini Tube Rotator at 10 rpm. The beads were recovered with a magnetic particle concentrator (Dynal® MPC, Invitrogen, Carlsbad, CA, USA) for 2 min. The supernatant was carefully removed, the pellet rinsed with 200 μL of 70 % ethanol and the process was repeated. Finally, the tubes were air-dried leaving the caps open, 100 μL of nuclease-free water were added and the beads were separated again until the supernatant was clear.

#### 2.3.4 Thermal lysis with “glass milk” purification

The “glass milk” protocol was based on the one described by Page et al. (Page et al., 2022a, 2022b) with slight modifications. The pellet was resuspended in 100 μL of nuclease-free water and 100 μL of a 4 % SDS solution. The suspension was incubated for 5 min at 99 °C and 1400 rpm in a thermomixer. After the lysis, 400 μL of 100 % isopropanol, 200 μL of 1.25 M NaCl, and 10 μL of “glass milk” were added. The mixture was incubated for 5 min at room temperature, then spun for 15 s, the supernatant was decanted, and the pellet was resuspended in 500 μL of 70 % ethanol, this step was performed twice. After the second pellet rinse, the samples were spun for 30 s, and the excess of ethanol was carefully removed with a pipette. Finally, the samples were air dried at 65 °C for 5 min with the leads open, and the pellet was resuspended in 100 μL of nuclease-free water to release the DNA from the silica, the suspension was spun for 15 s.

### 2.4 Real-time and colorimetric LAMP assays

#### 2.4.1 Primer design

Specific primers targeting the genes *stx*1 and *stx*2 were designed with PrimerExplorer V5 (https://primerexplorer.jp/e/index.html). To this end, GenBank references M19473.1 and X07865.1 were chosen for *stx*1 and *stx*2 respectively. Due to the existing variations within each of these genes, additional references were retrieved from GenBank (NZ_CP008957, AY170851, H19BSLT, Z36901.1 and M19473 for stx1 and AJ010730, AF043627, AY286000, DQ059012, L11079, Z37725, X07865, M21534, NZ_CP008957 for *stx*2), aligned with Geneious Prime® software Version 2023.1.1 (Biomatters Ltd., Auckland, New Zealand), and the position of each primer was confirmed. Primer sequences are provided in Table 2.

#### 2.4.2 Real-time LAMP

The new *stx*1/2 LAMP assays were optimized (simplex assays) taking advantage of fluorescence real-time LAMP, following the procedure described by Roumani et al. (Roumani et al., 2021a, 2021b), see Supporting Information Figures S1A to S1F and S2A to S2E. The final reaction volume was 25 μL composed of 15 μL of GspSSD2.0 Fast Isothermal Master Mix (ISO-004, OptiGene Ltd., Horsham, UK), 0.04 μL of ROX as a passive Reference Dye (Invitrogen™, Carlsbad, CA, USA), 1% Pullulan (TCI Europe, Zwinjdrecht, Belgium), 1 μL of *stx*1 and *stx*2 25X primer stock (multiplex, or STEC LAMP assay, see Table 2 for detailed primer concentration) and 5 μL of template DNA, the remaining volume was filled with sterile milliQ water. The reactions were run at 66 °C for 30 min with fluorescence acquisition every 30 s (60 cycles) in a QuantStudio™ 5 System and analyzed with QuantStudio™ Design & Analysis Software v1.5.1 (Applied Biosystems™, Foster City, CA, USA). Results confirmation was accomplished by performing a melt curve analysis, which consisted in heating at 95 °C for 1 s, 85 °C for 20 s, and heating again up to 95 °C with temperature increments of 0.015 °C and fluorescence acquisition during the process.

#### 2.4.3 Colorimetric LAMP

In regards to the colorimetric assay, two different strategies were used to determine which one could be better suited for field applications. In both cases, the final reactions included the primers for the detection of both *stx*1 and *stx*2 (multiplex). The first colorimetric approach was based on the addition, post-amplification of an intercalating dye, from now on SYBR-LAMP or SYBR, and the second one taking advantage of a commercial formulation, from now on Commercial Colorimetric LAMP or CC/CC-LAMP. The SYBR-LAMP was performed directly using the same parameters, and master mix, as in real-time, without the reference dye, and for the naked-eye detection the strategy described by Lamas et al. and Sukphattanaudomchoke et al. (Lamas et al., 2023; Sukphattanaudomchoke et al., 2020) 1 μL of SYBR Green I or SYBR Gold 1000X (Invitrogen™, ThermoFisher Scientific, Waltham, Massachusetts, USA), dissolved in DMSO, was placed on the lid and the tubes partially covered by Parafilm®, to avoid the dye from dropping into the reaction, and once the amplification time was completed, the tubes were vigorously vortexed to mix the amplicons with the dye and then visualized under white and UV light. For the latter an economic, <50€, nail curing lamp was used (emission wavelength 390 nm, ref: DR-618). Alternatively, for the CC-LAMP, the Visual detection RT Isothermal Master Mix (ISO-010RT-VIS, OptiGene Ltd., Horsham, UK) was also tested for which the only modifications from the real-time LAMP protocol were the removal of the reference dye and the increase in the incubation time up to 60 min for a better color discrimination. Both assays were performed in a Veriti™ Thermal Cycler (Applied Biosystems™, Foster City, CA, USA).

#### 2.4.4 Inclusivity/exclusivity

The inclusivity/exclusivity of the *stx* LAMP assays was initially assessed by performing nucleotide BLAST (https://blast.ncbi.nlm.nih.gov/Blast.cgi?PROGRAM=blastn&PAGE_TYPE=BlastSearch&LINK_LOC=blasthome) for each one of the primers. The results were confirmed *in vitro* by real-time LAMP in simplex and multiplex. The bacterial strains detailed in Table 1 were tested, these included target, *stx*-positive, and non-target, *stx-*negative (non-*E.coli* and *E. coli* strains from potentially pathogenic serogroups lacking the *stx* genes).

**Table 1:** Strain list and LAMP result.

Species	Source	Serotype	*stx* subtype	*stx*1	*stx*2	Species	Source	Serotype	*stx* subtype	*stx*1	*stx2*
*E. coli*	WDCM 00014	O157:H7	–	–	–	*E. coli*	FVL 468	O113:H21	*stx*2c	–	+
	CECT 5947	O157:H7	–	–	–		FVL 469	O166:H28	*stx*1c	+	+
	C179-12	O104:H4	–	–	–		FVL 470	O26:H11	*stx*1a	+	–
	T4/97	O157	*stx*2f	–	+		FVL 471	O146:H21	*stx*1c/*stx*2b	+	+
	LMV_E_2	O26	U	+	+		FVL 472	O157:H7	*stx*1a/*stx*2c	+	+
	LMV_E_3	O111	U	–	–		AMC 76	O157:H7	U	+	+
	LMV_E_4	O145	*stx*1a	+	–	*L. monocytogenes*	WDCM 00021	4b	–	–	–
	LMV_E_7	O103	U	–	+	*L. innocua*	CUP 1375	6a	–	–	–
	EF129	O45	–	–	–	*Salmonella* spp.	AMC 84	Wentworth	–	–	–
	FVL 461	O26:H11	*stx*1a	+	–		WDCM 00031	Typhimurium	–	–	–
	FVL 463	O146:H21	*stx*1c/*stx*2b	+	+		UB S1400	Enteritidis	–	–	–
	FVL 465	O5:HNM	*stx*1a/*stx*2a	+	+	*K. pneumoniae*	CECT 7787	–	–	–	–
	FVL 466	O103:H2	*stx*1a	+	–	*S. aureus*	WDCM 00034	–	–	–	–
	FVL 467	O111:HNM	*stx*1a	+	–	*S. agalactiae*	CECT 183	–	–	–	–

WDCM: World Data Centre for Microorganisms reference. ATCC: American Type Culture Collection. CECT: Spanish Type Culture Collection. LMV_E strains, along with EF129 and T4/97 were supplied by the National Institute for Agricultural and Veterinary Research (INIAV). AMC strains belong to the Collection from the Institute of Applied Microbiology – ASMECRUZ. UB: University of Bristol. CUP: Catholic University of Porto. FVL: strains provided by the *E. coli* Reference Laboratory (LREC) of the University of Santiago de Compostela (USC), Spain. “U”: Unknown *stx* subtype.

**Table 2:** STEC LAMP and multiplex qPCR primers and probes.

Primer	Sequence 5’ → 3′	Concentration (nM)	Modifications	Reference
stx1_F3	ACC ACG TTA CAG CGT GTT G	200	–	This study
stx1_B3	GCC CAC TGA GAT CAT CAA GT	200	–	
stx1_FIP	GTG AGG TTC CGC TAT GCG ACA T *tttt* ATC AGT CGT ACG GGG ATG C	1000	–	
stx1_BIP	GAC GCA GTC TGT GGC AAG AGC *tttt* TCC CCT CTG TAT TTG CCG AA	1000	–	
stx1_LF	AGA AGT AGT CAA CGA ATG GCG ATT T	300	–	
stx1_LB	ACG GTT TGT TAC TGT GAC AGC	300	–	
stx2_F3	AAT GGA GTT CAG TGG TAA TAC AAT G	200	–	
stx2_B3	CCA CTC TGA CAC CAT CCT CT	200	–	
stx2_FIP	TCT GCC TGA AGC GTA AGG CTT C *tttt* GAT GCA TCC AGA GCA GTT CT	1000	–	
stx2_BIP	CTG CTC CTG TGT ATA CGA TGA CGC C *tttt* CCC GAT ACT CCG GAA GCA CAT TGC	1000	–	
stx2_LF	TGC TGT GAC AGT GAC AAA ACG	400	–	
stx2_LB	AGA CGT GGA CCT CAC TCT G	400	–	
O157-rfbE-F	TCA ACA GTC TTG TAC AAG TCC AC	200	–	(Alejandro Garrido-Maestu et al., 2020)
O157-rfbE-R	ACT GGC CTT GTT TCG ATG AG	200	–	
O157-rfbE-P	AC TAG GAC CGC AGA GGA AAG AGA GGA A	150	Cy5/IAbRQSp	
eae-P3F	TGA CGG TAG TTC ACT GGA CTT C	200	–	Costa-Ribeiro et al. (2023)
eae-P3R	TGA CCC GCA CCT AAA TTT GC	200	–	
eae-P3P	TGG TCA GGT CGG AGC GCG TTA CA	150	TexRd-XN/IAbRQSp	
stx1-P3F	TGT CGC ATA GTG GAA CCT CAC	200	–	
stx1-P3R	CAG CTG TCA CAG TAA CAA ACC G	200	–	
stx1-P3P	ACG CAG TCT//GTG GCA AGA GCG ATG T	150	FAM/ZEN/IABkFQ	
stx2-P3F	AAC GGT TTC CAT GAC AAC GG	200	–	
stx2-P3R	CAG TGA GTG ACG ACT GAT TTG C	200	–	
stx2-P3P	TGC AAC GTG TCG CAG CGC TGG	150	ATTO550N/IAbRQSp	
NC-IAC-F	AGT TGC ACA CAG TTA GTT CGA G	100	–	(Garrido-Maestu et al., 2019)
NC-IAC-R	TGG AGT GCT GGA CGA TTT GAA G	100	–	
IAC-P	AGT GGC GGT//GAC ACT GTT GAC CT	100	YY/ZEN/IABkFQ	(A Garrido-Maestu et al., 2018)

The “*tttt*” is a linker among F2/B2 and their corresponding F1c and B1c as recommended by Lamas et al. (Lamas et al., 2023). YY (Yakima Yellow), IABkFQ (Iowa Black®FQ), IAbRQSp (Iowa Black®Sp) and ZEN (secondary, internal quencher) are trademarks from IDT.

#### 2.4.5 Dynamic range

The DNA from a fresh AMC 76 culture was obtained, and this was ten-fold serially diluted in nuclease-free water and the dilutions were analyzed following the three detection strategies mentioned, the real-time, SYBR, and CC LAMP assays, implementing the primers for each gene separately and combined, in order to assess the dynamic range, the analytical sensitivity and the potential effect the combination of the primers may have in the final result.

### 2.5 Reference STEC qPCR

To serve as the reference method, the multiplex qPCR assay described by Costa-Ribeiro et al. was selected (Costa-Ribeiro et al., 2023a, 2023b). The final primer and probe concentration is detailed in Table 2, other than these, the reactions were performed in a final volume of 20 μL with 10 μL TaqMan® Fast Advanced Master Mix (Applied Biosystems™, Foster City, CA, USA), 3 μL of template DNA and the remaining volume was filled with nuclease-free water. The reactions were run in a QuantStudio™ 5 System as described previously. Samples providing a positive result were confirmed by plating in Chrom as described previously.

### 2.6 Determination of the Limit Of Detection (LOD)

To calculate the LOD the method reported by Wilrich & Wilrich, and indicated by NordVal, was used (NordVal, 2017; Wilrich and Wilrich, 2009). To determine the LOD, samples spiked with decreasing concentrations are needed so that a level is reached with positive and negative results, and this allows the model to make the statistical determination of the LOD. In this sense, the LOD50 was calculated being this the concentration at which one has a 50 % probability of getting a positive result (NordVal, 2017). In this study, 11 samples were analyzed with the three different LAMP assays in the multiplex format (combining *stx*1 and *stx*2 primers in the same reaction).

### 2.7 Fitness-for-purpose

The LOD50 calculated as described in M&M 2.6 was used as the cut-off of each LAMP assay so that only samples spiked above this level were considered. Every sample was initially classified as being a Positive or Negative Agreement (PA/NA), Positive or Negative Deviations (PD/ND) attending to the definitions set by NordVal (2017). In a subsequent step, the deviations were re-classified as False Positive or Negative (FP/FN), and True Positive (TP) after result confirmation, once more as defined by NordVal.

The samples classified in this way were used to determine the relative sensitivity (SE), specificity (SP), and accuracy (AC) of the method, in addition to the Cohen's k (k) to determine the degree of agreement with the expected results. These parameters were calculated following the formulae reported by Anderson et al. and Tomás et al. (Anderson et al., 2011; NordVal, 2017; Tomás et al., 2009).

### 2.8 Statistical analysis

Graphical representation, and statistical analyses, of the data were performed with GraphPad Prism version 8.0.0 for Windows (GraphPad Software, San Diego, California USA, www.graphpad.com). Data comparison was performed with one-way, or two-way, ANOVA and Tukey post-hoc with a significance value of *p* < 0.05.

## 3 Results

### 3.1 Evaluation of the DNA extraction protocols

**Fig. 1:**
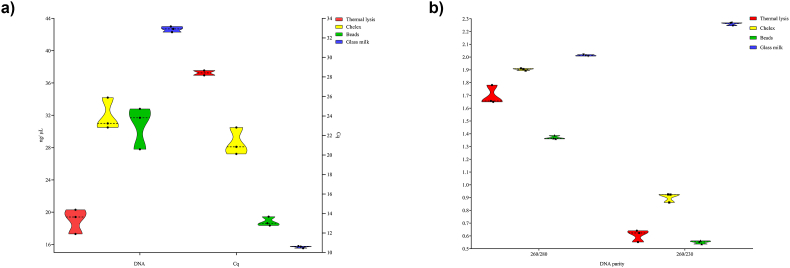
DNA concentration and Cq values obtained with real-time LAMP A) and DNA purity absorbance ratios B) obtained for the four DNA extraction protocols evaluated.

Four simple DNA extraction protocols, potentially suitable for field applications, were evaluated. These were 1) thermal lysis, 2) chelex, 3) thermal with magnetic bead purification, or 4) glass milk purification. For all the parameters evaluated, DNA concentration, purity, and performance in real-time LAMP, the thermal lysis with glass milk purification outperformed the other three. The statistical analyses indicated that the glass milk protocol obtained significantly higher DNA concentration, and significantly lower Cq value, likewise this protocol obtained statistically the highest purity ratios out of the 4 protocols tested, see Fig. 1A and B.

These DNA extracts were also analyzed by multiplex qPCR confirming the previous observation where the thermal lysis coupled with the glass milk purification outperformed the other protocols. In addition to this observation, it was also noted that the direct thermal lysis and the chelex protocols were not capable of removing qPCR inhibitory compounds resulting in reaction inhibition; while with real-time LAMP, only 1 out of the 3 samples extracted by direct thermal lysis showed inhibition, being possible to detect both genes in the other 2 samples and in all 3 extracted with chelex.

### 3.2 Inclusivity/exclusivity

**Fig. 2:**
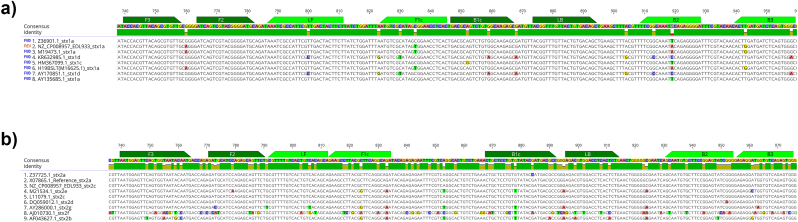
Sequence alignment and position of LAMP primers targeting *stx*1 A) and *stx*2 B).

Additional sequences, to those used for primer design, were included in the final alignment to better asses potential hybridization issues related to sequence variation due to *stx*1 and *stx*2 subtypes. These sequences were retrieved from NCBI. The alignment of the newly designed primers with the panel of reference sequences selected did not show any potential limitation as, if existing, nucleotide variations were not present in critical places, i.e. 5′ and 3’ ends, see Fig. 2A–B where the corresponding *stx* subtype is also presented. This observation was further confirmed by BLAST, as only *stx* sequences were identified.

The step for the evaluation of the inclusivity/exclusivity of the novel assays consisted of *in vitro* analyses of pure bacterial cultures. To this end, a total of 28 strains covering 7 different bacterial species were included. In the selected panel, 20 different *E. coli* strains were included, and covered 11 different serogroups (O157, O26, O111, O146, O103, O45, O146, O5, O104, O113, and O166) which were gathered from official culture collections, as well as from natural origin, see Table 1. Among the different *E. coli* strains included, 5 were only positive for *stx*1, 3 only for *stx*2, 7 were positive for both genes, and 5 were negative for *stx*1 and 2. Other than these, 3 different serovars of *Salmonella enterica* were analyzed along with 1 *L. monocytogenes,* 1 *L. innocua*, 1 *S. aureus*, 1 *S. agalactiae,* and 1 *K. pneumoniae*, being all these negative for *stx*1 and *stx*2.

The analysis of pure cultures allowed us to experimentally determine the Tm for each target. In this sense, the Tm for *stx*1 was determined to be 87.18 ± 0.18 °C while for *stx*2 was 87.84 ± 0.32 °C and 87.58 ± 0.38 °C when both primer sets were mixed for the simultaneous detection of both genes, see Supporting information Figs. S3A and S3B.

### 3.3 Dynamic range

**Fig. 3:**
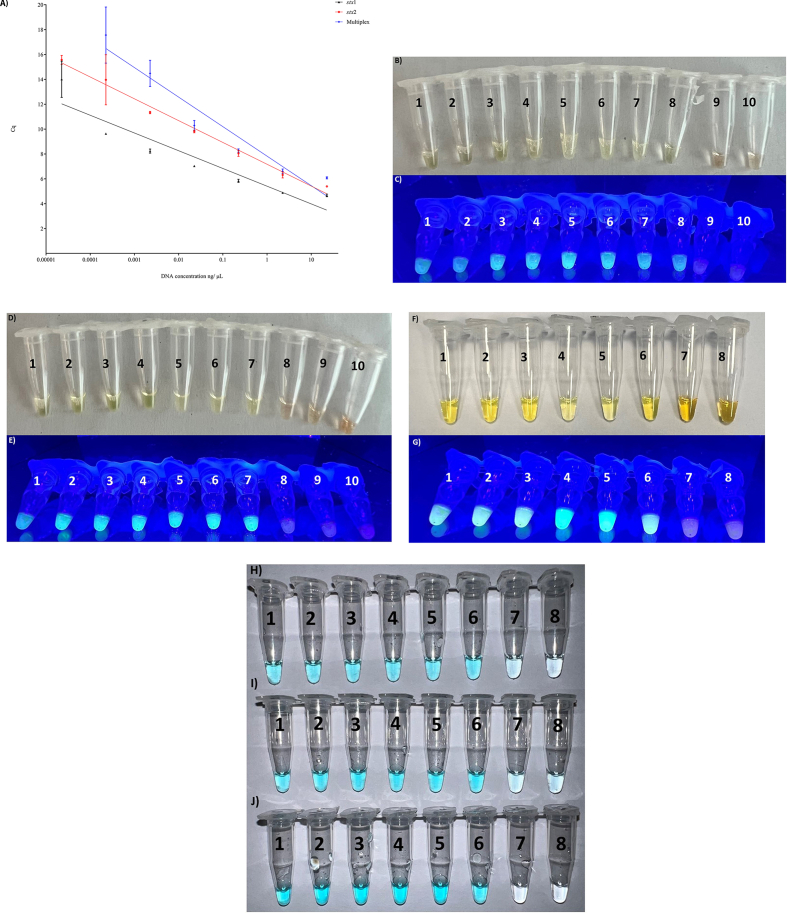
Dynamic range with pure DNA ten-fold serially diluted from 22.6 ng/μL down to 0.00226 pg/μL analyzed with real-time LAMP A), *stx*1 with SYBR under white light B), *stx*1 with SYBR under UV light C), *stx*2 with SYBR under white light D), *stx*2 with SYBR under UV light E), *stx*1 and *stx*2 with SYBR under white light F) *stx*1 and *stx*2 with SYBR under UV light G), *stx*1 with CC H), *stx*2 with CC I) and *stx*1 and *stx*2 with CC J).

The evaluation of the dynamic range, and analytical sensitivity, was assessed with all three detection strategies, namely, real-time, SYBR, and Commercial LAMP for each one of the genes separately and combined. The range covered was from 22.6 ng/μL to 0.00226 pg/μL, an 8 log dynamic range, see Fig. 3A. The lowest concentration was only reachable with the real-time LAMP, even though for *stx*1 only 2 out of the 3 replicates were positive at the lowest concentration (all positive targeting *stx*2). In a similar way by mixing both sets of primers, only 1 out of 3 replicates were positive with 0.00226 pg/μL and 0.0226 pg/μL.

When the analysis was done with the colorimetric strategies, the dynamic range was reduced to 6 logs as it was only possible to detect down to 0.0226 pg/μL regardless of the naked-eye detection approach selected, and regardless of whether the assays were performed in simplex or multiplex, see Fig. 3C–D.

### 3.4 Determination of the LOD

**Fig. 4:**
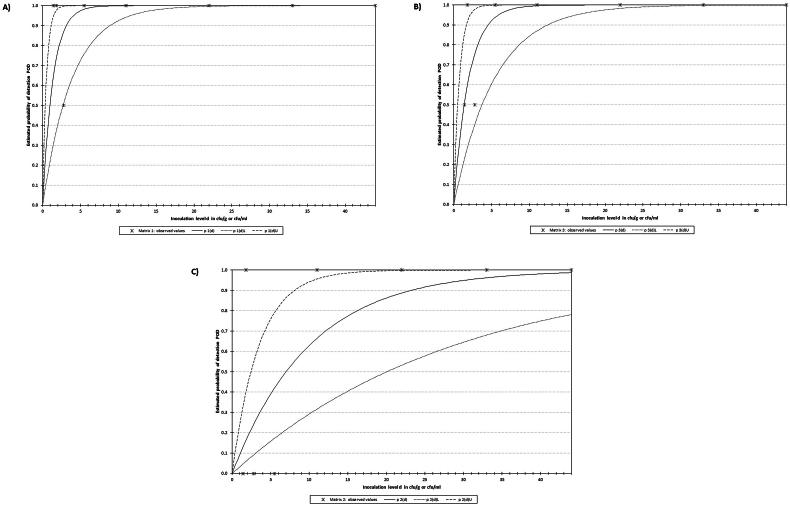
Graphical representation of the data for the determination of the LOD50. Data retrieved from the mathematical model described by Wilrich and Wilrich (2009). In the graphs, “p 1(d)" represents the probability of detection (POD), “p 1(d)U" and “p 1(d)L" are the Upper and Lower limits with 95 % confidence, respectively, and the "*" indicate the data experimentally obtained. Data from real-time LAMP A), SYBR B), and Commercial Colorimetric C).

A total of 11 samples spiked with 8 different concentrations were analyzed following the new method. The spiking levels ranged from 44 down to 1.4 CFU/25 g. The model determined that the LOD50 was 0.9, 1.4, and 7.0 CFU/25 g for the real-time, SYBR and CC LAMP assays. In line with what was previously commented, the real-time assay, which had the lowest analytical sensitivity, reached the lowest LOD50. These results are graphically depicted in Fig. 4A to C.

### 3.5 Fitness-for-purpose

Overall, a total of 25 samples were included in the current study, 11 spiked and 14 non-inoculated. Due to the low LOD50 reached by the real-time assay, 0.9 CFU/25 g, all of them were considered for the evaluation of this assay. Only 1 ND was observed in a sample spiked with 2.8 CFU/25 g as it was positive by qPCR; however, as this result was not confirmed by plating on Chrom, the sample was re-classified as an NA. Thus, SE, SP, and AC values were of 100.0 % and a Cohen's k of 1.00.

When focusing on the SYBR assay, the LOD50 was 1.4 CFU/25 g, and so all 25 samples were included in the evaluation. In this sense, the same sample as of the real-time assay was negative, likewise, the ND was re-classified as a NA due to the lack of confirmation on Chrom. In addition to this, one sample more inoculated with 1.4 CFU/25 g, was also negative, in this case, typical colonies were observed on Chrom and so, the sample was re-classified as an FN. This observation resulted in a SE of 90.9 %, SP of 100.0 %, and AC of 96.0 % with a Cohen's k of 0.92.

Lastly, the CC LAMP, which was calculated to have the highest LOD50, 7.0 CFU/25 g. This resulted in the non-consideration of 7 samples below the LOD50. Out of the remaining, no deviations were observed, thus achieving SE, SP, and AC values of 100.0 % along with a Cohen's k of 1.00. Detailed information about the inoculation pattern is provided in Table 3, and Table 4 summary of the performance of each one of the assays reported is provided.

**Table 3:** Results obtained by STEC LAMP and qPCR with spiked samples.

Concentration (CFU/25 g)	N	Real-time		SYBR		CC		qPCR		Chrom	
		+	–	+	–	+	–	+	–	+	–
44.0	1	1	0	1	0	1	0	1	0	1	0
33.0	1	1	0	1	0	1	0	1	0	1	0
22.0	1	1	0	1	0	1	0	1	0	1	0
11.0	1	1	0	1	0	1	0	1	0	1	0
5.5	2	2	0	2	0	0	2	2	0	2	0
2.8	2	1	1	1	1	0	2	2	0	1	1
1.8	1	1	0	1	0	0	1	1	0	1	0
1.4	2	2	0	1	1	0	2	2	0	1	1
0	14	0	14	0	14	0	14	0	14	0	14

N: Total number of samples inoculated with the indicated concentration. “+” and “-“denote the number of samples providing a positive and negative respectively with each assay.

**Table 4:** Evaluation of the STEC LAMP method with the different detection strategies.

Assay	LOD50	N	PA	NA	PD	ND	FN	TP	FP	SE	SP	AC	k
Real-time	0.9	25	10	15	0	1*	0	0	0	100.0	100.0	100.0	1.00
SYBR	1.4	25	10	14	0	1*	1	0	0	90.9	100.0	96.0	0.92
CC	7.0	18	4	14	0	0	0	0	0	100.0	100.0	100.0	1.00

N: number of samples. LOD50: Limit of Detection in CFU/25 g. PA: Positive Agreement. PD: Positive Deviation. NA: Negative Agreement. ND: Negative Deviation. FN: False Negative. TP: True Positive. FP: False Positive. *Samples re-classified upon confirmation on selective agar media. SE: relative sensitivity. SP: relative specificity. AC: relative accuracy. k: Cohen's kappa, interpreted as “almost complete concordance” (0.81–1.00) according to previous references (DG, 1991). “Real-time”: real-time fluorescence monitoring LAMP, “SYBR”: naked-eye color change observation based on the addition of SYBR Green, “CC”: naked-eye color change observation based on a commercial colorimetric master mix.

## 4 Discussion

The goal of the current study was to develop a PON method for the identification of the presence of STEC in RTE vegetables. To this end, we took advantage of LAMP and evaluated its performance with three different detection chemistries, real-time fluorescence monitoring, end-point colorimetric observation after the addition of SYBR Green, and a commercial colorimetric formulation. Other methods have been reported for the detection of STEC performing real-time fluorescence, or turbidity, tracking (Wang et al., 2014), as well as end-point naked eye observation of color change upon the addition of SYBR Green (Baraily et al., 2019; Zhao et al., 2013), other components such as hydroxynaphthol blue (Xia et al., 2022) or even commercial preparations (Sarah Azinheiro et al., 2022). However, many of these methods are either performed in well-equipped laboratories or directly oversee the need to process the sample to reach detectable bacterial levels and remove LAMP-inhibitory compounds. In the present study, in addition to developing a novel LAMP assay, particular care was taken with the pre-LAMP, sample processing steps. In this sense, the sample dilution factor was reduced to increase the bacterial concentration, four simple DNA extraction protocols were compared, and three different LAMP detection strategies were evaluated to provide a simple, economical, and reliable method, suitable to be performed at PON, in a decentralized setup with low resources, being this the major novelty of the reported study.

A set of newly designed primers was used for the detection of *stx*1 and *stx*2. Attending to the *in silico* analyses performed, BLAST and alignment of different variants of these genes, as well as the *in vitro* tests with STEC strains of different origins and serogroups, both assays were specific for their corresponding target. It was observed that extending the amplification time may lead to some cross-reactivity among both genes when highly concentrated, pure DNA was used as the template. This phenomenon was previously reported in other LAMP assays (Kaur et al., 2023; Tomlinson, 2013; Y. Zhang and Tanner, 2022). Given the fact that this only occurred among *stx*-positive strains and that the method was focused on the detection of STEC, and ultimately it will implement both sets of primers in the same reaction, it was considered a minor issue. Other than this, simplex assays demonstrated to have a wide dynamic range and being capable of reaching very low bacterial DNA concentration both, in simplex and multiplex, regardless of the detection chemistry selected. In this sense, the most sensitive strategies were the real-time and SYBR LAMPs in their simplex format, regardless of the target in agreement with previous studies where real-time and naked-eye color observation were compared (Li et al., 2023; Xie et al., 2022). Contrary to this, the implementation of the commercial formulation was 10 times less sensitive, this most likely is associated with the actual dye implemented in the commercial product, and so highlights the importance of properly selecting the detection chemistry depending on the needs and purpose of the method (Alejandro Garrido-Maestu and Prado, 2022; J. Zhang et al., 2022). When the dynamic range, and the analytical sensitivity, were evaluated in the assay combining both sets of primers, a 1 log reduction was observed, this may be the result of a certain degree of competition among the primers as a total of twelve were implemented in a single reaction. This would be in line with what Fan et al. already reported (Fan et al., 2018). Overall, the slight decrease in analytical sensitivity was not considered a limiting factor as by implementing a suitable enrichment step, along with an efficient DNA extraction protocol, high bacterial DNA is assured as previously reported by D'Agostino et al. when dealing with the detection of *Salmonella* spp. (D'Agostino et al., 2015) and Garrido-Maestu et al. targeting *L. monocytogenes* (A Garrido-Maestu et al., 2018).

Considering what was commented previously, the design of a new analytical assay by itself results of limited interest. For this reason, a suitable DNA extraction protocol was also pursued considering the inherent problematic associated to vegetables in terms of inhibitory compounds such as chlorophylls, and polysaccharides, among others (Lalonde and Gajadhar, 2016; Schrader et al., 2012). The robustness of LAMP against this type of compounds was confirmed as the reported assays returned positive results, with a certain delay, even after a simple thermal lysis, or chelex treatment, while the reference qPCR failed in line with what was previously reported for PCR/qPCR assays (Kaneko et al., 2007; Yang et al., 2014). Then, the implementation of a DNA purification, and concentration, step was pursued. In this regard, these are straightforward ways to go, and as expected, they returned the best results out of the protocols tested. Both strategies presented pros and cons, for instance the magnetic beads only need a magnetic stand to be performed, along with some ethanol, but the beads must be stored at 4 °C to assure their stability; while the glass milk protocol relies on a centrifuge, but the chemicals needed are fairly inexpensive and be stored at room temperature. Considering the specific features of each protocol, along with amplification data retrieved, and that the beads protocol returned more variable Tm values as shown in Supporting information Fig. S4, most likely associated to the presence of residual ethanol in the final extract, in agreement with the observations of Bonner et al. (Bonner & Klibanov, 2000), the glass milk was considered to be the best option of its combination with the STEC LAMP assay. In subsequent experiments, it was confirmed that the selected protocol provided DNA with enough quality and quantity, to be detected with the three strategies tested, namely real-time, SYBR, and CC LAMP as well as by qPCR. The glass milk protocol obtained similar results, or even better, in terms of DNA purity (A260/280 and A260/230) as those of other previously reported commercial kits as the ratios of 260/280 were ∼2.0 while the 260/230 were ∼2.2, both within the optimal ranges of pure DNA (Geuther, 2007), as depicted in Fig. 1B (Desneux and Pourcher, 2014; A. Garrido-Maestu et al., 2015; Santos et al., 2015).

Lastly, once the optimal molecular protocol was established, it was applied to spiked commercial RTE vegetable samples purchased from local suppliers. In order to improve the performance of the method an enrichment step in mTSBn, as described by the STEC ISO standard was included (ISO/TS, 2012). The only modification was the reduction of the dilution factor which allows for a reduction in the cost of the assay, the space needed in an incubator per sample, reduces the time needed to reach the optimal temperature during the incubation stage. Implementing the enrichment step, it was possible to reach an LOD50 below 10 CFU/25 g regardless of the detection approach selected for the STEC LAMP however, those implementing fluorescence, either in real-time or SYBR, were determined to have an LOD50 below 2 CFU/25 g. These results are in line with the evaluation of the analytical sensitivity where also the lowest concentration, 0.0022 pg/μL was reachable with the real-time assay, regardless of whether it was performed targeting one gene or both in the same reaction. The LOD50 values obtained were very similar to those previously reported for LAMP, and qPCR-based methods, targeting STEC or other foodborne pathogens (Alejandro Garrido-Maestu et al., 2020; Lamas et al., 2023; Sohrabi et al., 2022; Yu et al., 2020). Even though it was not part of the focus of the current study, following the reported methodology improved the LOD of the qPCR assay set as a reference, 8.7 CFU/25 g, as all the samples resulted positive, including those spiked with 1.4 CFU/25 g, in agreement with previous studies which reported higher sensitivity of qPCR-based methods compared to LAMP (Deguo et al., 2008; A Garrido-Maestu et al., 2018; Roumani et al., 2021a, 2021b). It must be noted that the original qPCR study was focused on same-day detection thus was possible to be performed in only 5 h.

The fact that the real-time and SYBR assays outperformed the CC was expected as both rely on the same amplification master mix. Thus, similar results were likely to be obtained, being the addition of external SYBR the only factor potentially causing differences among them. In the present study, when testing the SYBR assay, it was decided to place the dye in the inner part of the lid of the tubes, and partially covered with parafilm to avoid it from dropping during the amplification. This procedure was initially described by Sukphattanaudomchoke et al., and later on, confirmed to be effective by Lamas et al. (Lamas et al., 2023; Sukphattanaudomchoke et al., 2020). Keeping the dye out of the reaction was important because, at the concentration needed to generate a naked-eye color change, it would have resulted in reaction inhibition (Oscorbin et al., 2016; Quyen et al., 2019). It was confirmed that the dye could be added directly to the reaction after the amplification; however, it was discarded as the tubes will have to be opened, and it has been extensively discouraged in the literature due to the very high likelihood of contamination of the laboratory, what will ultimately increase the false positive samples due to this cross-contamination (Amin Almasi, 2012; Chen et al., 2023; Hanyue et al., 2023; Karthik et al., 2014; N. B. Quoc et al., 2021; Nguyen Bao Quoc et al., 2018).

Lastly, when the performance of the method was evaluated, regardless of the detection chemistry selected, all the parameters returned values higher than 90 % with no major deviations. More specifically, with the real-time assay there was only one ND in a sample spiked with 2.8 CFU/25 g, which was positive by qPCR however, no typical colonies were isolated in the confirmation step thus the sample was re-classified as an NA and so, a 100.0 % was calculated for the SE, SP and AC of this specific assay with a Cohen's k of 1.00 which translates as “almost complete concordance” (DG, 1991). When focusing on the SYBR assay, the same sample as of the real-time, was also negative, and likewise, the ND resulted in an NA due to the lack of typical colonies however another deviation was observed in this case a sample spiked even with a lower concentration, 1.4 CFU/25 g, and in this case the qPCR-positive results was confirmed on Chrom and so this ND was reclassified as an FN what resulted in a decrease in the SE (90.9 %) and the AC (96.0 %) while the SP remained (100.0 %) and an overall Cohen's k of 0.92, once more being interpreted as “almost complete concordance”; it is worth noting that this particular sample was spiked at a very low concentration and right at the LOD level thus, being these factors behind the FN result obtained in line with what was previously reported by Garrido-Maestu et al. (Alejandro Garrido-Maestu et al., 2018). For the remaining LAMP assay, the CC, in line with what happened with the real-time, no deviations were observed and so the SE, SP, and AC values were 100 %, and the Cohen's k 1.00; but it must be kept in mind that the LOD was significantly higher, 7 CFU/25 g, compared to the other two assays. All these results are in agreement with those previously reported for LAMP-based methods intended for the detection of different foodborne pathogens (S Azinheiro et al., 2022; Bella et al., 2021; D'Agostino et al., 2015) and even qPCR (Bouvier et al., 2023; Cloke et al., 2016; Fachmann et al., 2017; Margot et al., 2013).

Considering what has been reported, a novel STEC LAMP method, suitable for its implementation in low-resource setups has been successfully developed. The fact that this new method implements a well-established enrichment procedure allows for a reduction in its cost and acceptability by the end users as, if needed, the ISO standard may be performed for confirmation/comparison purposes. In addition to this, the implementation of the glass milk DNA extraction protocol has been demonstrated to be a simple, inexpensive, and efficient way of obtaining the nucleic acids needed for the LAMP assay. These two previous steps together allowed for the reliable detection of STEC in RTE salad samples by LAMP with three different detection strategies once more suitable for decentralized setups as miniaturized devices and systems are already available in the market to perform the reported assays, i.e. the Genie II and III or the BioRanger for real-time fluorescence monitoring (Diaz et al., 2019; Higgins and Smith, 2020; Ibarra-Meneses et al., 2018), for the SYBR assay a simple heat source as the thermomixers already used for the DNA extraction, a miniaturized PCR heat block (Rubinfien et al., 2020) may be used along with a handheld UV lamp or even non-specialized UV device like the one reported herein (Carvalho et al., 2022; Lamas et al., 2023) along with other types of systems such as the Bento Lab which implements a centrifuge, PCR block and transilluminator (Toppings et al., 2021; Werner et al., 2022) may be used; and, obviously, the CC assay, which even though was outperformed by the previous two assays still returned good results for all the parameters tested, would only need the heat source for what any of the systems and devices just commented, could be used. Despite all the advantages reported, an effort to simplify the enrichment protocol must still be performed, and caution must always be taken to assure good laboratory practices to avoid reagent cross-contamination particularly when working in decentralized setups.

## 5 Conclusions

The novel STEC LAMP method reported here was developed, and evaluated, implementing three different detection strategies. Out of these, the selection of real-time fluorescence monitoring obtained the overall best results in terms of LOD, analytical sensitivity, and performance in spiked samples. However, two other naked-eye colorimetric alternatives, with excellent results, were also provided so that the final user may select among them depending on particular needs and equipment availability. The combination of these assays with an inexpensive, low-complexity DNA extraction protocol, along with a proper enrichment protocol, truly allows for this method to be deployed in laboratories with low resources and decentralized settings.

## CRediT authorship contribution statement

**Ana Costa-Ribeiro:** Investigation, revision. **Alexandre Lamas:** Writing – review & editing. **Azucena Mora:** Resources, revision. **Marta Prado:** Funding acquisition, Writing – review & editing. **Alejandro Garrido-Maestu:** Conceptualization, Methodology, Supervision, Validation, Writing – original draft.

## Declaration of competing interest

The authors declare that they have no known competing financial interests or personal relationships that could have appeared to influence the work reported in this paper.

## Data Availability

Data will be made available on request.
