# Supplementary material for: Moving towards on-site detection of Shiga toxin-producing Escherichia coli in ready-to-eat leafy greens
Source: Curr Res Food Sci. 2024 Mar 7;8:100716. doi: 10.1016/j.crfs.2024.100716 (PMC10950744; doi:10.1016/j.crfs.2024.100716)
Supplement: Multimedia component 1 [file mmc1.docx]

**Supporting Information**

**Moving towards on-site detection of Shiga toxin–producing *Escherichia coli* in ready-to-eat leafy greens**

Ana Costa-Ribeiro^1,2^, Alexandre Lamas^3^, Azucena Mora^4^, Marta Prado^1^, and Alejandro Garrido-Maestu^1,*^

^1^International Iberian Nanotechnology Laboratory, Av. Mestre José Veiga s/n, 4715-330 Braga, Portugal

^2^Department of Biochemistry, Genetics and Immunology, University of Vigo, 36310 Vigo, Spain

^3^Food Hygiene, Inspection and Control Laboratory (Lhica), Department of Analytical Chemistry, Nutrition and Bromatology, Veterinary School, Campus Terra, Universidade da Santiago de Compostela 27002, Lugo, España

^4^Laboratorio de Referencia de *E. coli* (LREC), Dpto. de Microbioloxía e Parasitoloxía, Facultade de Veterinaria, Universidade de Santiago de Compostela (USC), Lugo, Spain

^5^Instituto de Investigación Sanitaria de Santiago de Compostela (IDIS), Santiago, Spain

*Corresponding authors: [alejandro.garrido@inl.int](mailto:alejandro.garrido@inl.int)

Figures:


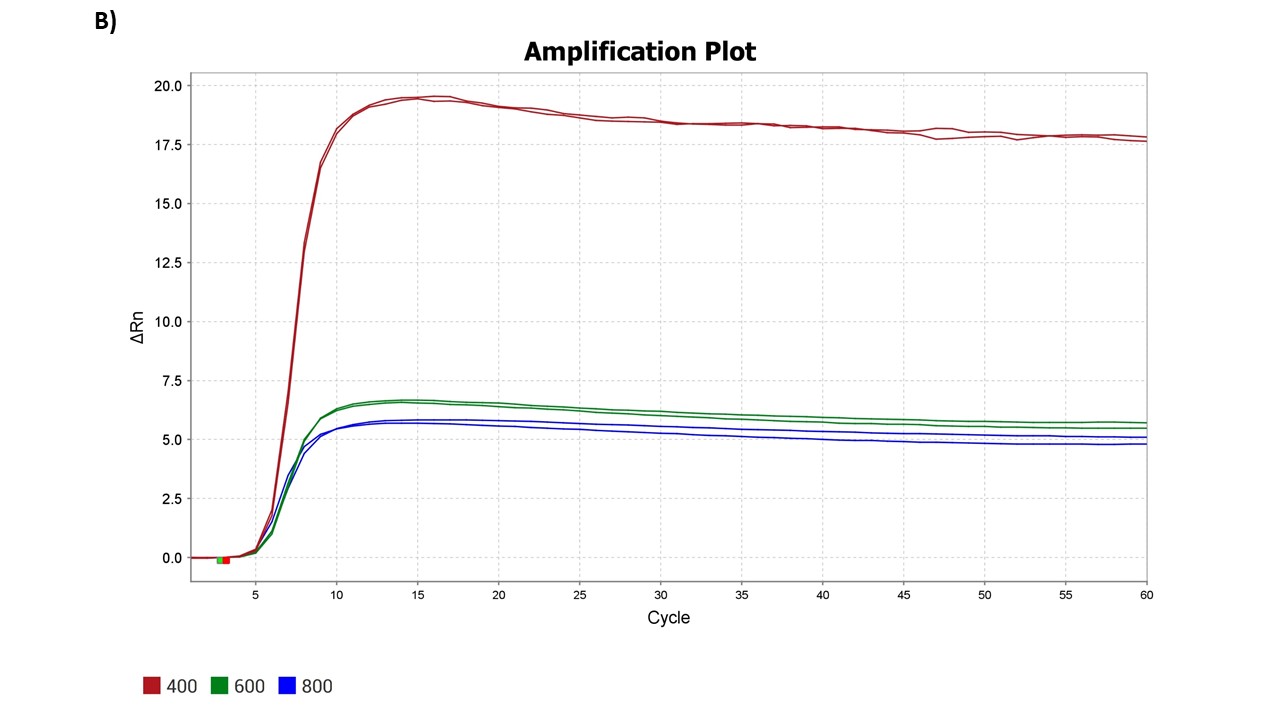

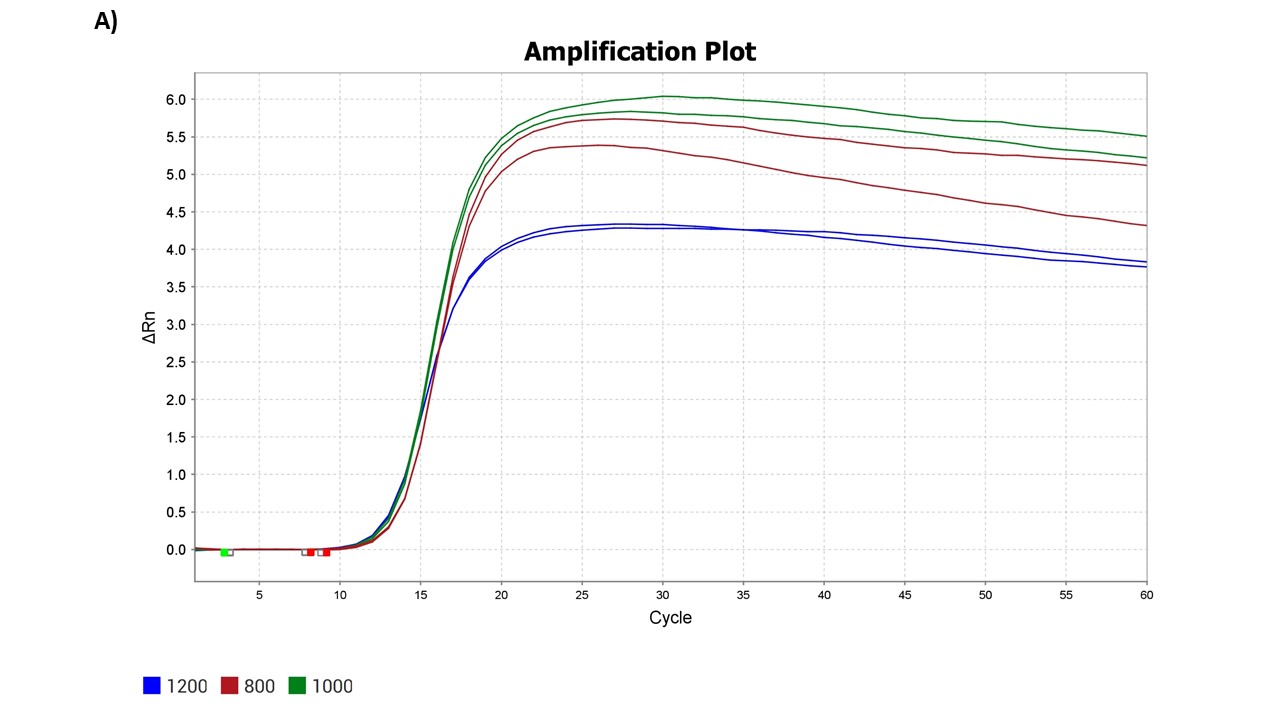

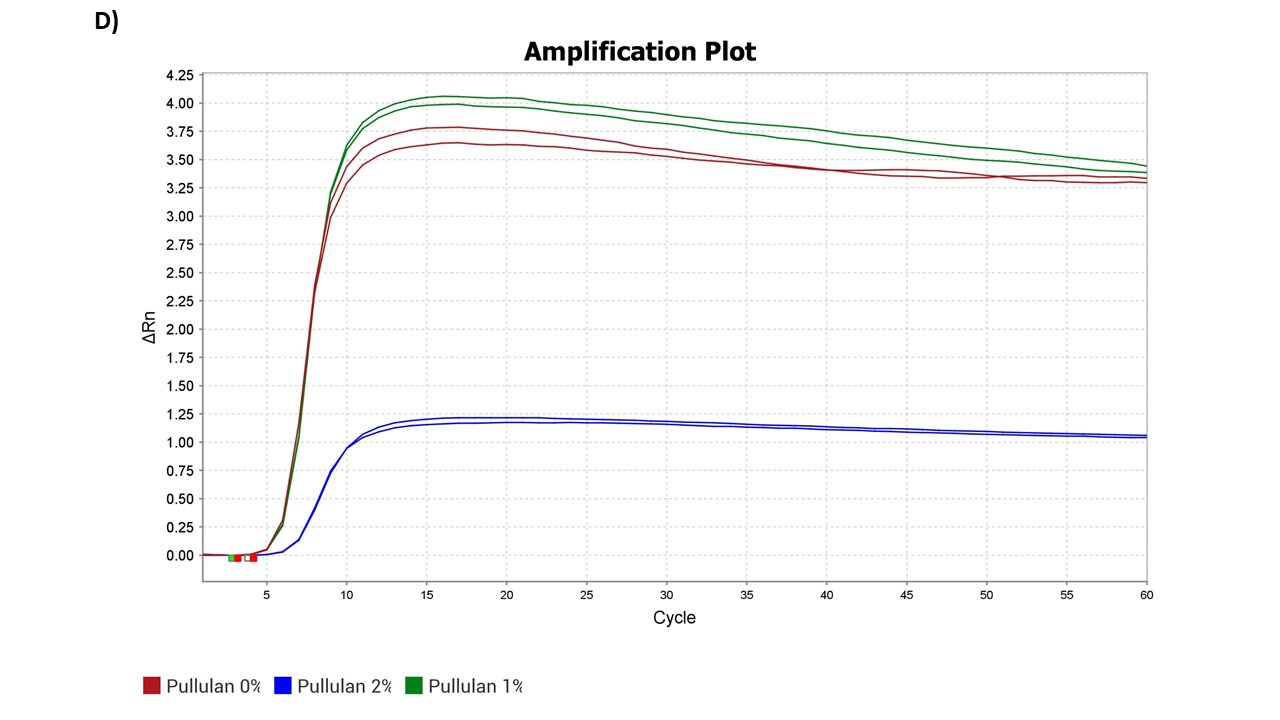

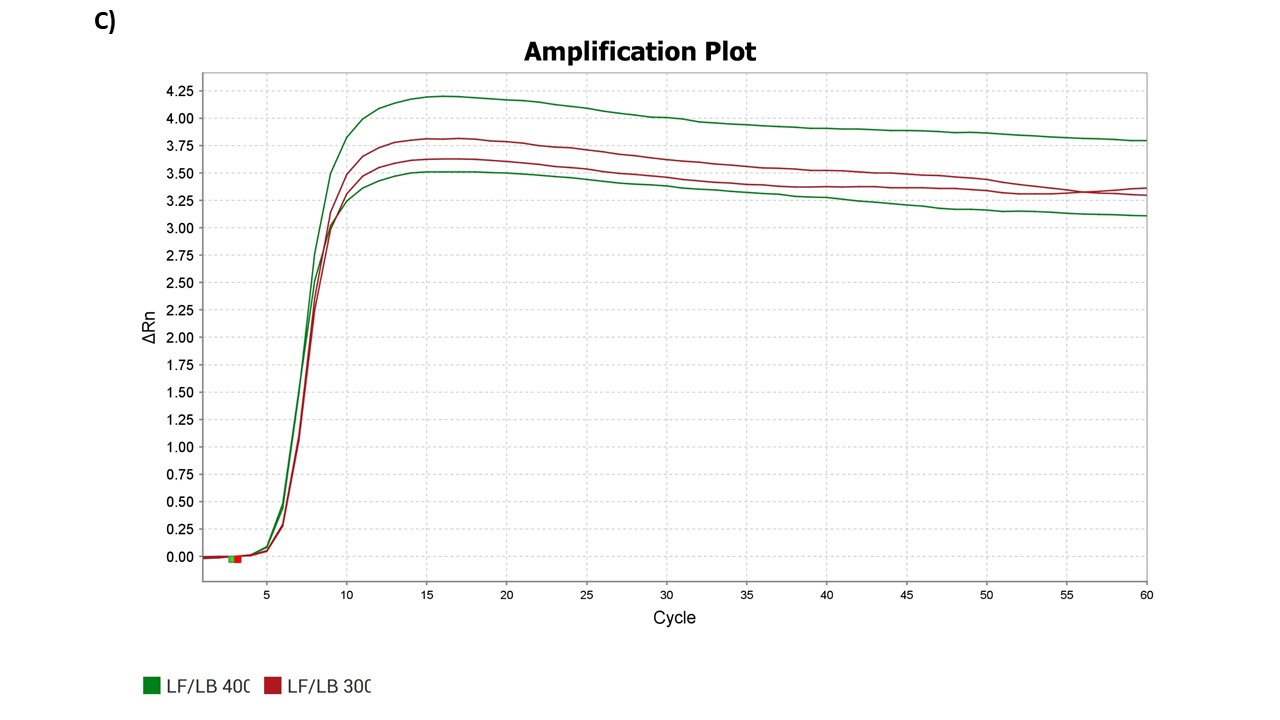

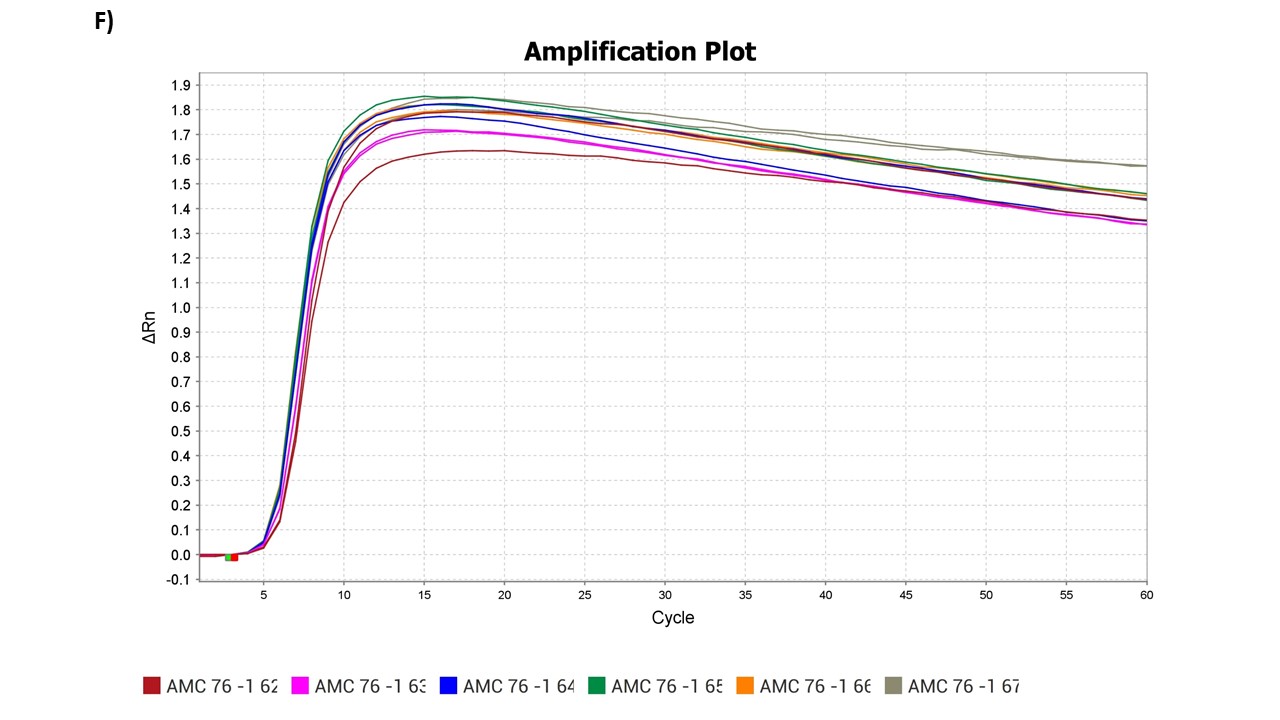

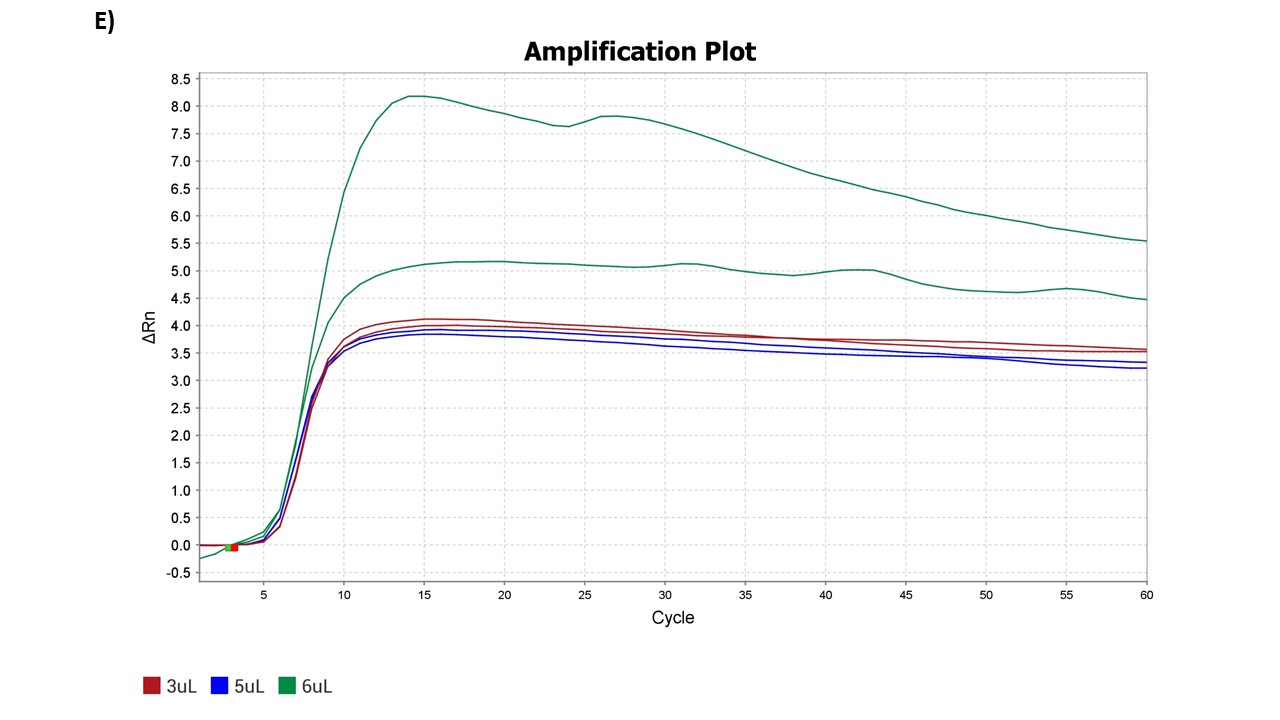


S1. Results obtained by real-time fluorescence LAMP over the optimization of *stx*1. Optimization of FIP/BIP concentration with 200 nM of F3/B3 A.) Optimization of LF/LB with 1000nM FIP/BIP and 200 nM F3/B3 in B) and C). Optimization of the concentration of pullulan with 1000nM FIP/BIP, 200 nM F3/B3 and 300 nM LF/LB D). Optimization of the volume of template DNA with 1000nM FIP/BIP, 200 nM F3/B3, 300 nM LF/LB and 1% pullulan E). Optimization of the amplification temperature with 1000nM FIP/BIP, 200 nM F3/B3, 300 nM LF/LB, 1% pullulan and 5 µL of template DNA F). After analysis, optimal temperature determined to be 66 °C


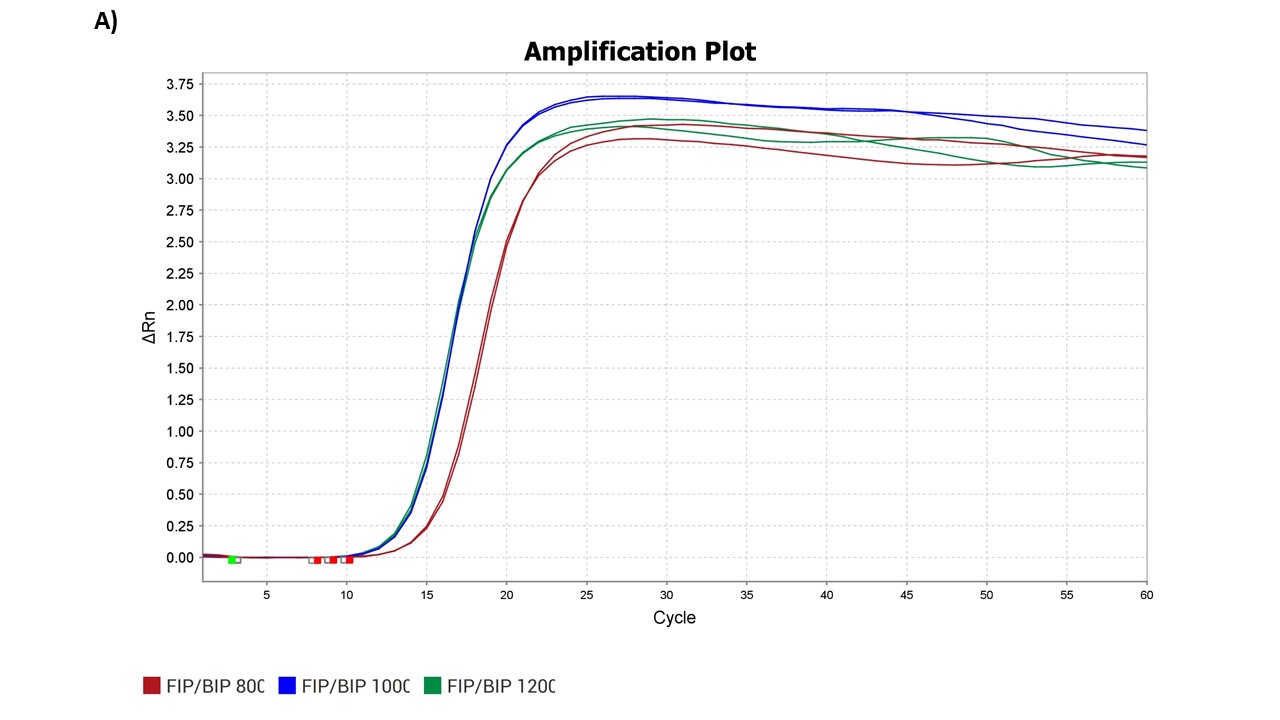

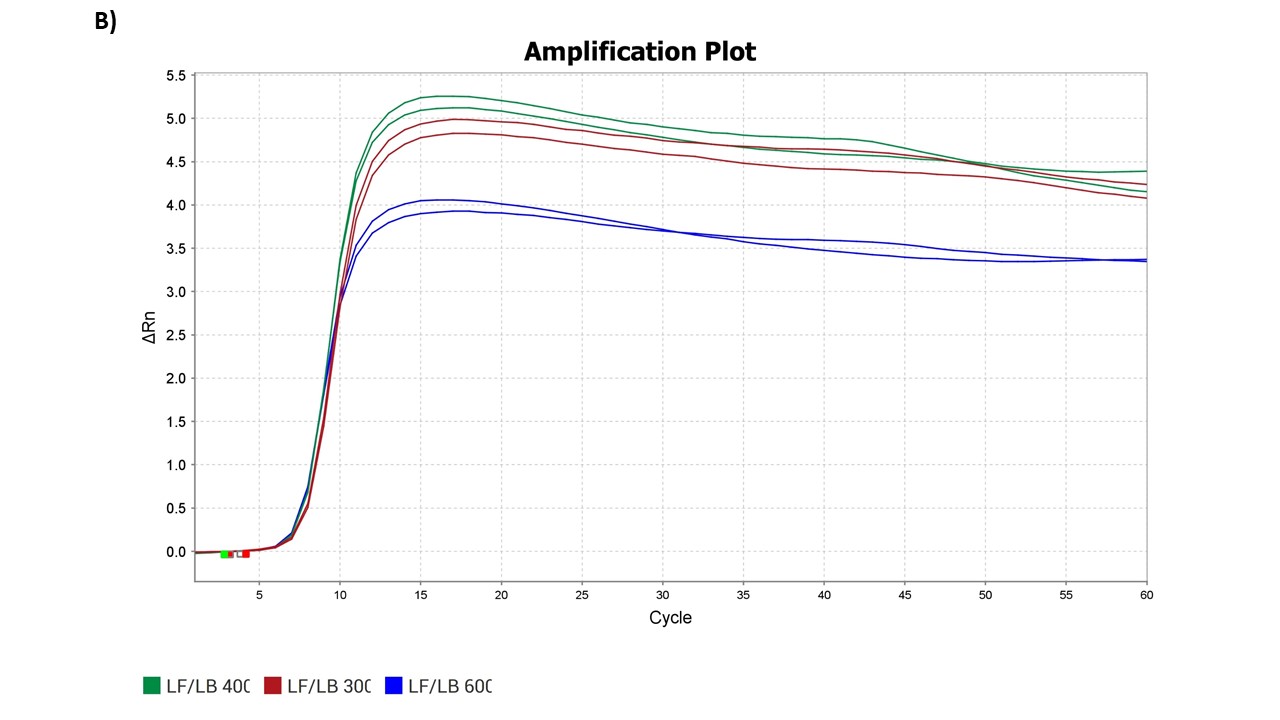

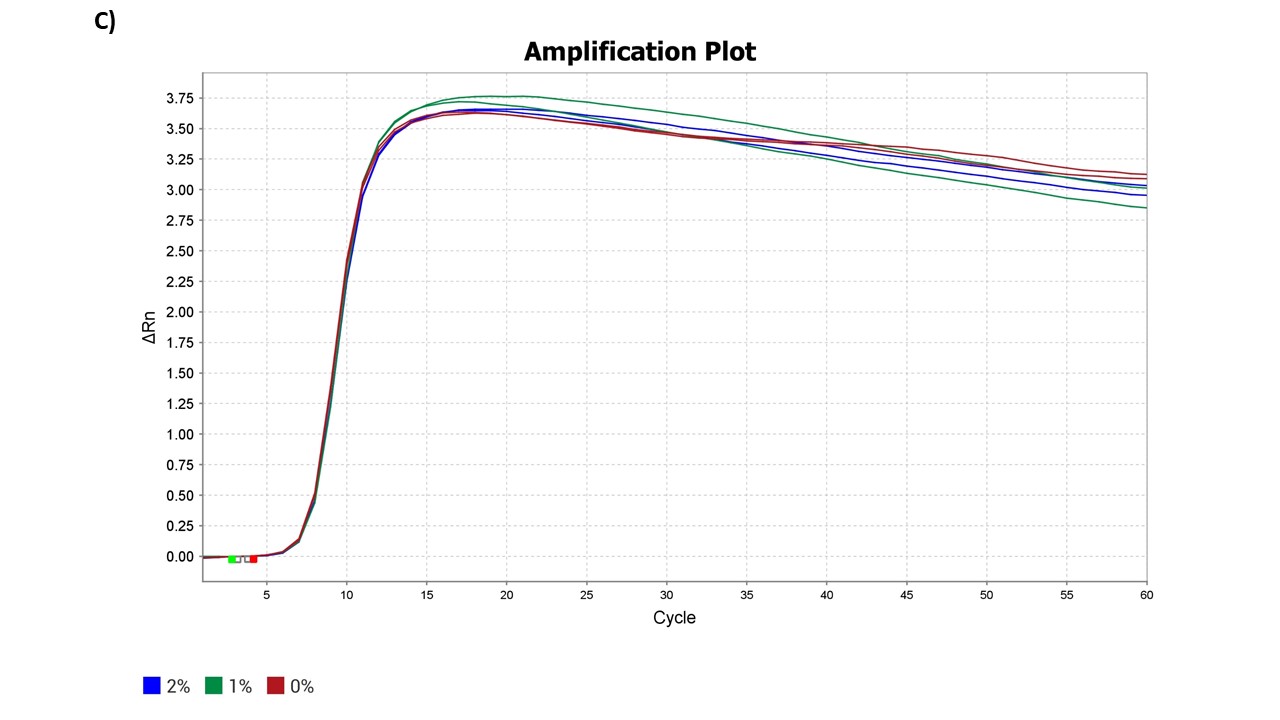

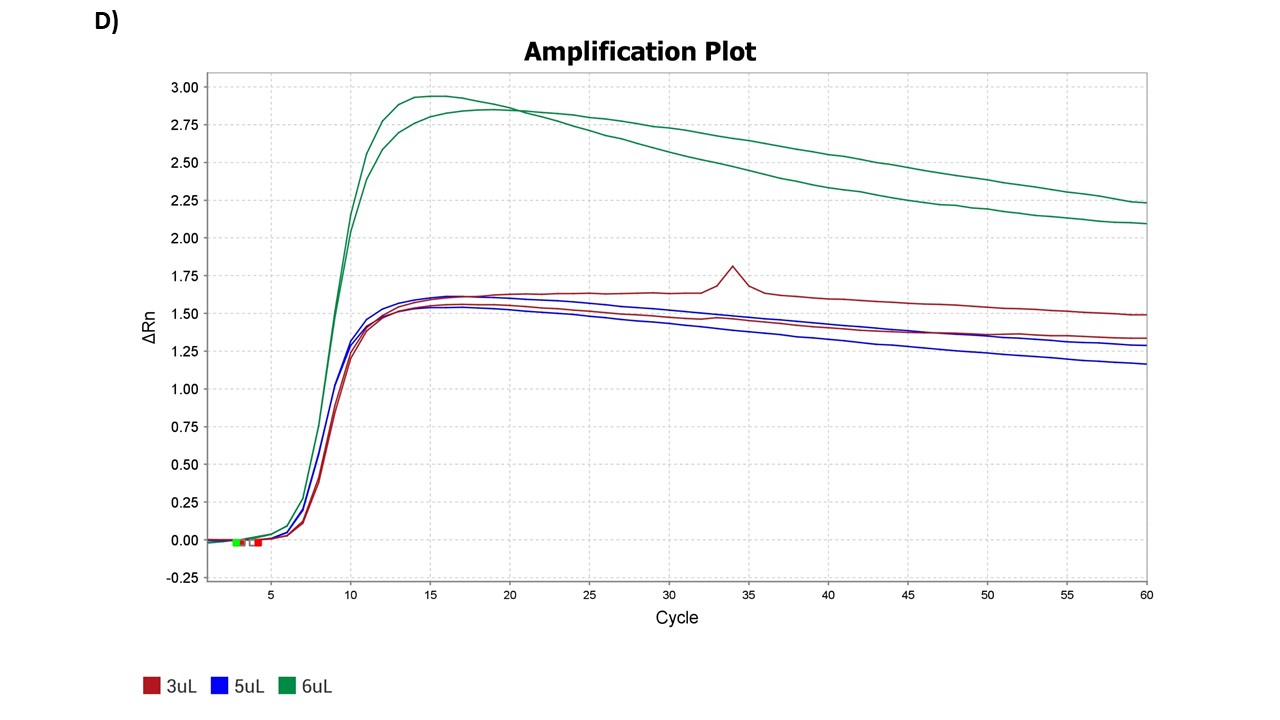


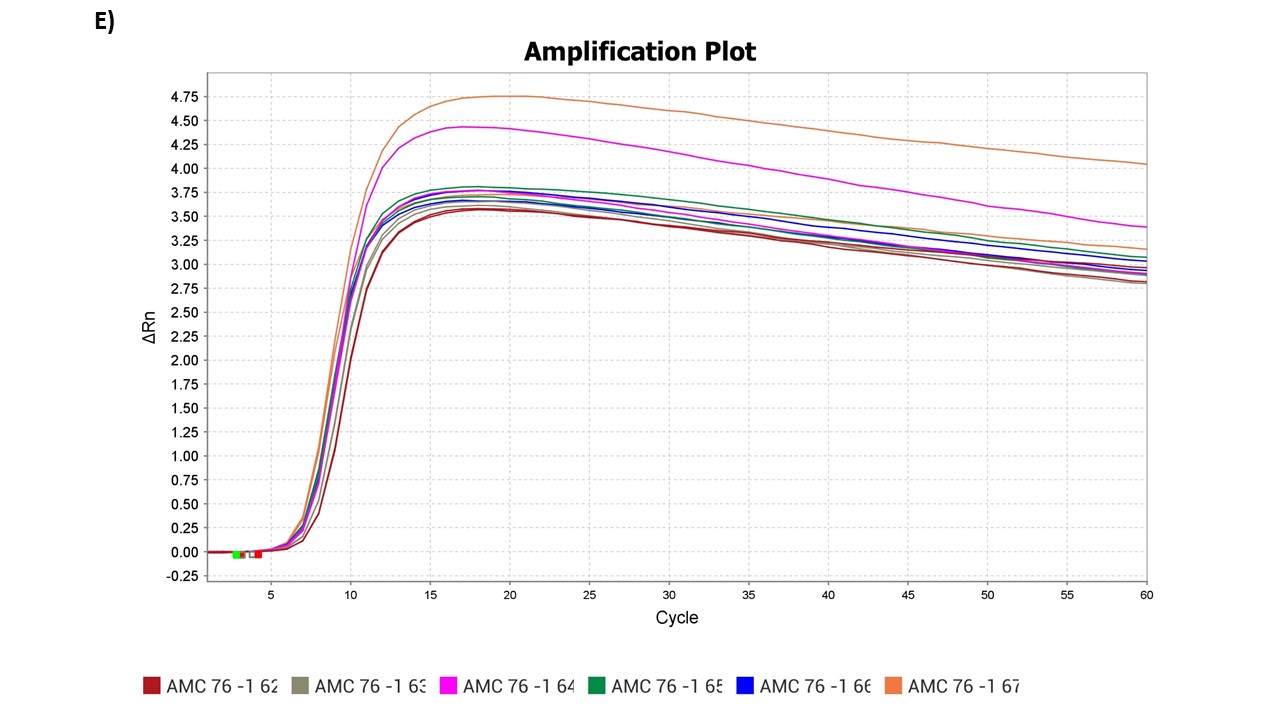


S2. Results obtained by real-time fluorescence LAMP over the optimization of *stx*2. Optimization of FIP/BIP concentration with 200 nM of F3/B3 A.) Optimization of LF/LB with 1000nM FIP/BIP and 200 nM F3/B3 in B). Optimization of the concentration of pullulan with 1000nM FIP/BIP, 200 nM F3/B3 and 400 nM LF/LB C). Optimization of the volume of template DNA with 1000nM FIP/BIP, 200 nM F3/B3, 300 nM LF/LB and 1% pullulan D). Optimization of the amplification temperature with 1000nM FIP/BIP, 200 nM F3/B3, 300 nM LF/LB, 1% pullulan and 6 µL of template DNA E). After analysis, optimal temperature determined to be 67 °C.

For the multiplex STEC LAMP the volume of template was unified for *stx*1 and *stx*2 to 5 µL, and the amplification temperature at 66 °C


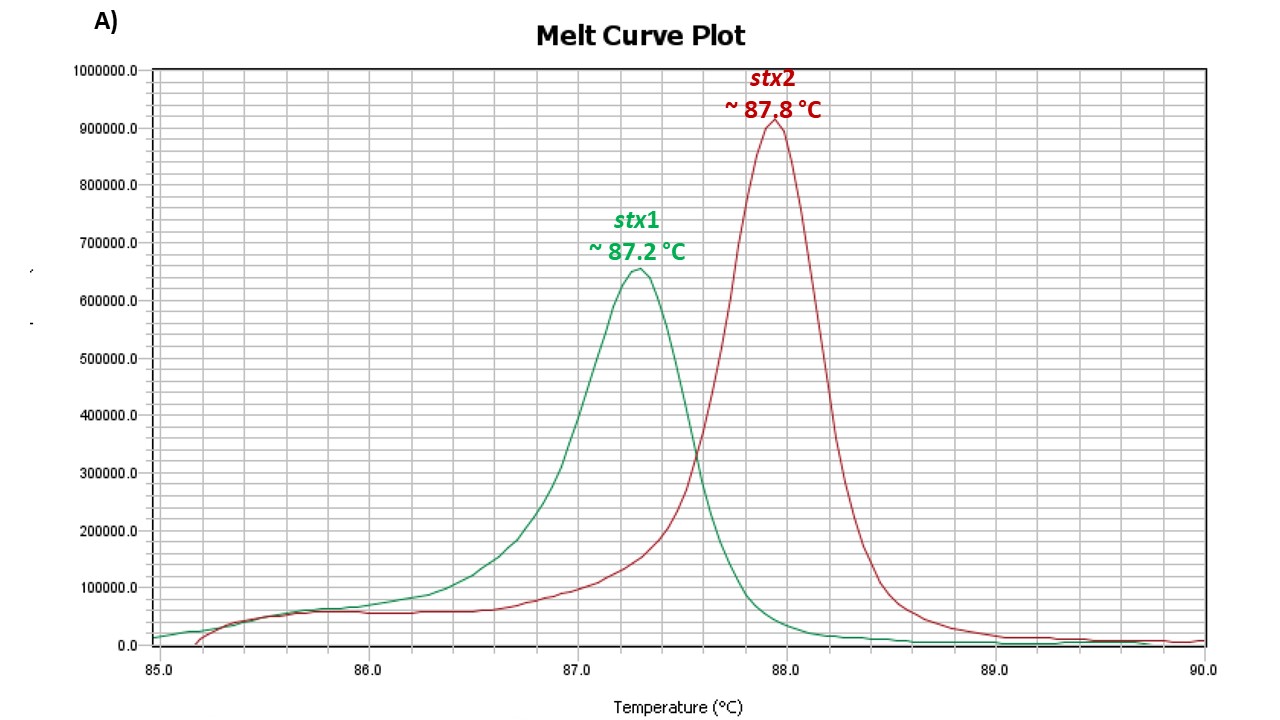

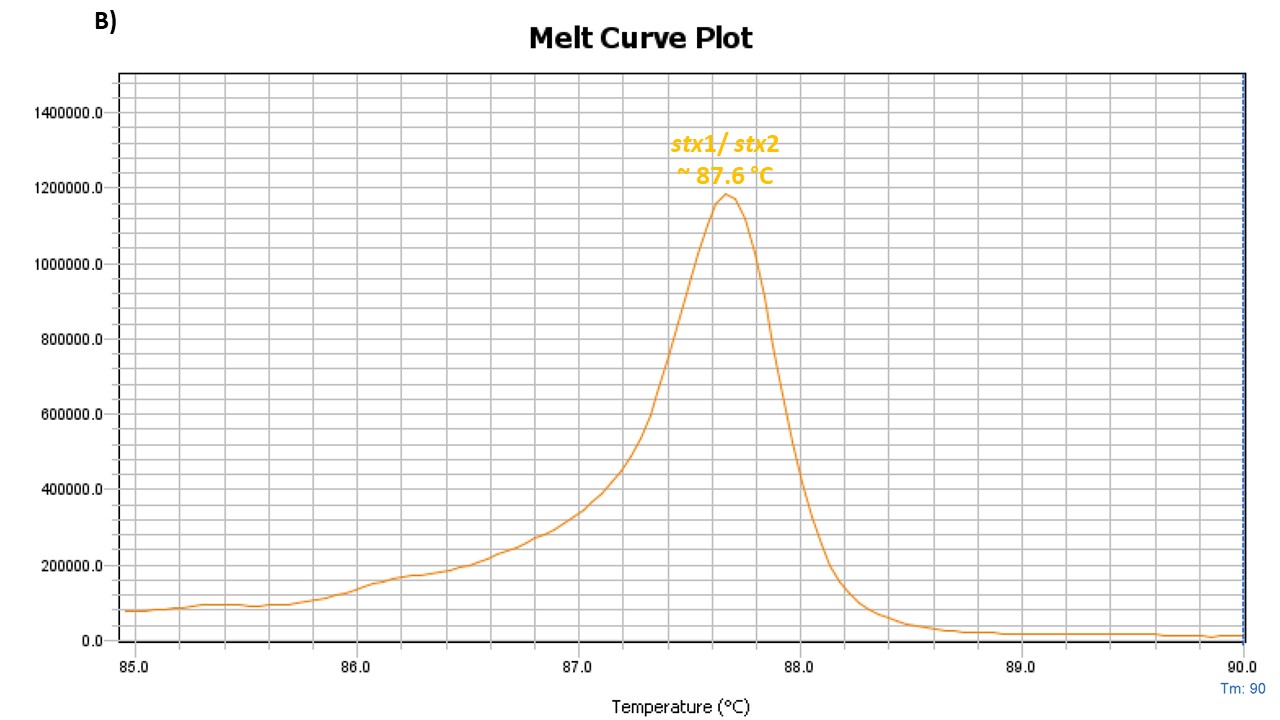


S3. Representative melt curves obtained by real-time fluorescence LAMP targeting *stx*1 and *stx*2 individually A) and *stx*1 and *stx*2 in multiplex B).


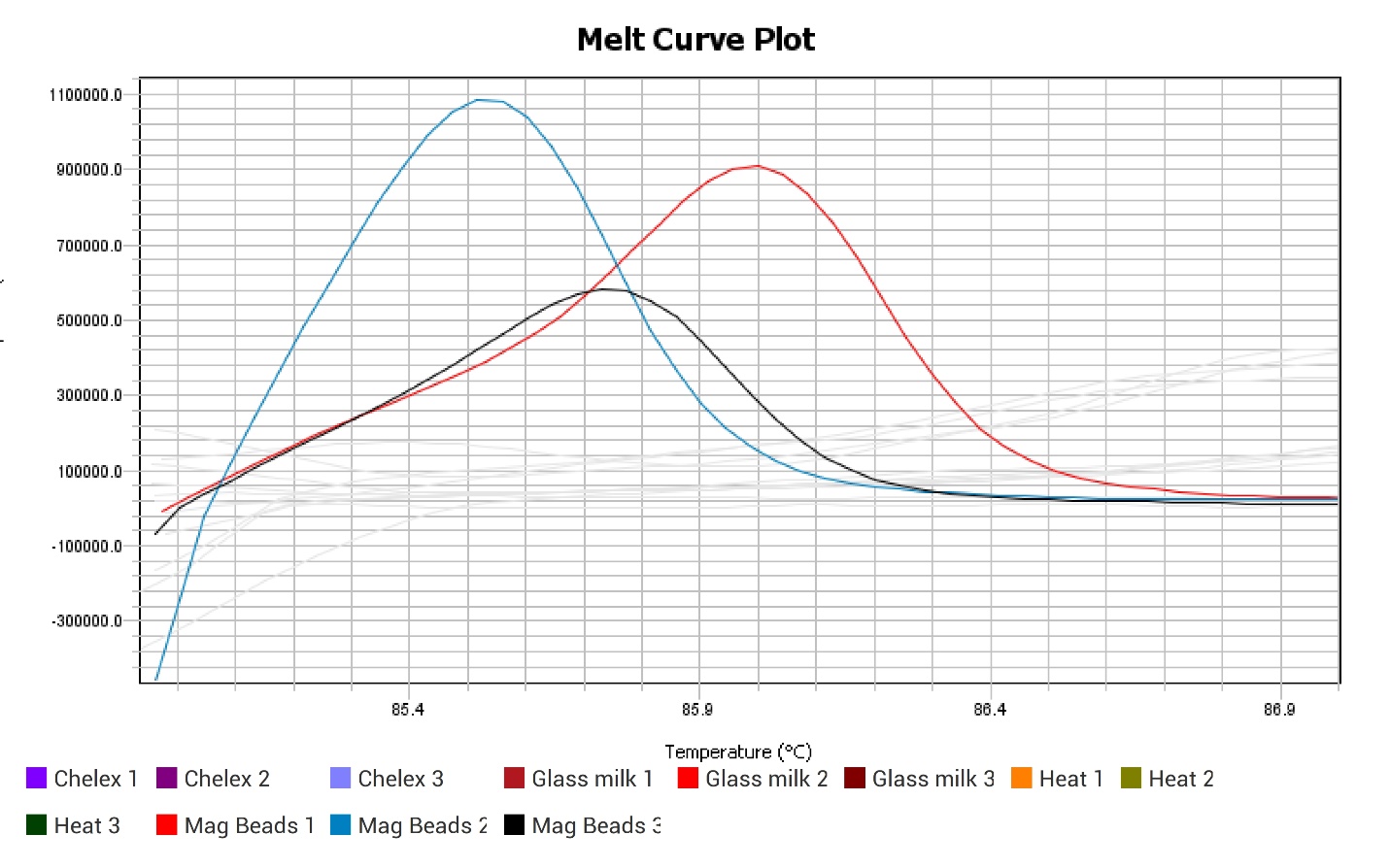


S4. Melting curves of three samples extracted with the magnetic beads protocol.
